# Evaluating the effectiveness of various teaching methods on dental plaque removal in children: a quasi-experimental study

**DOI:** 10.1186/s12887-025-05438-6

**Published:** 2025-02-12

**Authors:** Somayeh Khoramian Tusi, Zahra Momeni, Hajar Hamdollahpoor, Nastaran Parviz, Mahsa Ghorbani

**Affiliations:** 1 Department of Pediatric Dentistry, School of Dentistry, Alborz University of Medical Sciences, Karaj, Iran; 2 Department of Community Oral Health, School of Dentistry, Alborz University of Medical Sciences, Karaj, Iran; 3 Student Research Committee, Alborz University of Medical Sciences, Karaj, Iran

**Keywords:** Dental plaque index, Health education, Pediatric dentistry

## Abstract

**Objective:**

Dental plaque is a major contributor to oral diseases, particularly in children, but its impact can be significantly mitigated through targeted oral health education. This study aimed to evaluate the effectiveness of various tooth-brushing instructional methods in reducing dental plaque in children.

**Methods:**

A total of 120 children, aged 6 to 8, attending the pediatric department of Alborz Dental School, were randomly selected for the study. Participants were divided into four groups of 30 children each (15 boys and 15 girls). Each group received a different instructional method: (1) demonstration of tooth brushing on a dental model, (2) self-brushing in front of a mirror, (3) brushing another child’s teeth in front of a mirror, and (4) instruction via a standardized video. Dental plaque levels were measured using a disclosing agent before and after training, with a two-week follow-up to assess the impact of instruction. Senior students provided group-specific brushing training. Data were analyzed using SPSS-22 with ANOVA and t-tests, with significance set at *p* < 0.05.

**Results:**

All four groups demonstrated significant reductions in plaque levels post-training and at the two-week follow-up compared to baseline, as measured by the O’Leary index. The group trained with dental models showed the most substantial plaque reduction (*p* < 0.001).

**Conclusion:**

Instruction using dental models proved to be the most effective and sustainable method for reducing dental plaque in children, highlighting its potential for impactful and enduring oral hygiene education.

**Supplementary Information:**

The online version contains supplementary material available at 10.1186/s12887-025-05438-6.

## Introduction

Dental caries is one of the most prevalent chronic diseases in children [1]. Globally, between 60% and 90% of elementary-aged children are affected by dental caries [2]. According to the World Health Organization, approximately 514 million children suffer from tooth decay annually [3]. The incidence of caries is inversely related to age, with younger children being more vulnerable [4, 5]. Factors such as socioeconomic status, household acculturation, access to oral health resources, fluoride exposure, age, and geographic location significantly influence the prevalence of dental caries [6].

The primary cause of dental caries is the accumulation of microbial plaque on tooth surfaces. This plaque—a biofilm composed of oral bacteria, salivary proteins, and food particles—creates an environment conducive to acid production by pathogenic bacteria, leading to tooth decay. Notably, Streptococcus mutans, a bacterium commonly found in dental plaque, is highly tolerant of acidic conditions and is a key contributor to caries due to its prolific acid production [7].

Given the harmful effects of microbial plaque, early establishment of effective oral hygiene practices is critical. Teaching children proper tooth-brushing techniques forms the foundation for lifelong oral health [8]. Effective brushing depends on mastering the correct technique [9], and educational interventions play a pivotal role in motivating children to adopt better oral health habits. However, identifying the most effective instructional methods is essential for achieving optimal results [10].

The selection of an appropriate, high-impact teaching method is crucial for fostering lasting learning outcomes in children [11]. Evidence supports a variety of educational approaches—such as lectures, videos, and pamphlets—for significantly improving plaque scores [12]. Studies have shown that lecturing can be more effective than pamphlets [13], while combining oral instruction with demonstrations offers additional benefits, including enhanced knowledge and improved plaque control [14]. Innovative approaches like differential learning have demonstrated significant improvements in oral hygiene and follow-up outcomes compared to traditional methods [15]. Similarly, educational games can aid in refining tooth-brushing techniques [16], and motivation coupled with face-to-face instruction has proven especially effective in reducing plaque indices [17].

Numerous studies highlight the superiority of individualized instruction over audiovisual or child-model teaching methods [18, 19]. However, while the benefits of tooth-brushing instruction have been widely documented, few studies focus on identifying the most effective educational techniques [20, 21]. This study aims to evaluate the effectiveness of various tooth-brushing instructional techniques in reducing dental plaque in children, thereby contributing to the understanding and optimization of preventive oral health programs.

## Materials and methods

This quasi-experimental study was approved by the Ethics Board of the Medical Faculty at Alborz University of Medical Sciences, Karaj, Iran (approval code: IR.ABZUMS.REC.1398.061). All procedures were conducted following the principles outlined in the Declaration of Helsinki.

### Participants

A total of 120 children, aged 6–8, were selected through simple random sampling from those attending the pediatric department at Alborz Dental School during 2019–2020. Inclusion criteria required participants to be in good health, cooperative (Frankel behavior rating scale 3 or 4), and within the specified age range. Written consent was obtained from parents before participation. Children were excluded if they presented with systemic conditions (e.g., diabetes), had used antibiotics, NSAIDs, or mouthwash in the prior month, wore orthodontic appliances or space maintainers, experienced toothpaste sensitivity, or had cavitated or painful teeth. Participants or guardians who expressed dissatisfaction or whose conditions interfered with learning or tooth brushing were also excluded.

### Interventions

Tooth brushing was taught using a distinct instructional method for each group, with all participants trained in the standardized horizontal scrub technique, a method deemed appropriate for young learners [22, 23]. The teaching method for each group was determined randomly:

- Group 1: Instruction via demonstration on dental models.
- Group 2: Self-brushing instruction in front of a mirror.
- Group 3: Brushing instruction on another child’s teeth while positioned in front of a mirror.
- Group 4: Instruction through a standardized video tutorial (Available at: https://youtu.be/Cmzp1wdjaw).

These tailored, group-specific methods provided a structured approach for evaluating the effectiveness of different instructional techniques. Based on previous studies [20, 21, 24, 25] we hypothesized that group 1 (instruction via demonstration on dental models) would show the best results.

### Procedure

Before training, plaque levels were assessed using a disclosing agent applied by one of the authors (MG). Participants were then trained according to their group-specific method. All children were instructed to brush their teeth for two minutes twice daily using identical soft toothbrushes (Colgate Slimsoft, Colgate-Palmolive Company, USA) and toothpaste (Mild and Fresh, Bath, Iran), which were provided to ensure consistency. They had a chart to record their toothbrushing daily (supplementary file 1).

Plaque levels were reassessed immediately after training and again at a two-week follow-up. Examinations were conducted using disposable dental mirrors and disclosing agents, which stained plaque-affected areas. Surfaces with plaque were marked using a red pencil, while surfaces without plaque were left unmarked. Lost tooth surfaces were recorded with an “X” and excluded from calculations. The plaque index was calculated by dividing the total number of plaque-affected surfaces in the upper and lower jaws by the total number of surfaces, then multiplying by 100.

### Data recording

A standardized form was used to record demographic data and plaque indices at three stages: before training, after training, and two weeks post-training. Each tooth was divided into four surfaces for detailed analysis. The O’Leary plaque control index was used to assess the percentage of plaque-affected surfaces.

### Statistical analysis

Data were analyzed using SPSS-22 software (IBM SPSS Inc., Chicago, IL, USA). Descriptive statistics and measures of dispersion were calculated for data description and index comparison across the three stages. Paired-samples t-tests were conducted to compare pre- and post-training plaque indices within each group, while independent samples t-tests were used to assess differences between boys and girls. Group comparisons were performed using ANOVA, with statistical significance set at *p* ≤ 0.05.

## Results

**Fig. 1:**
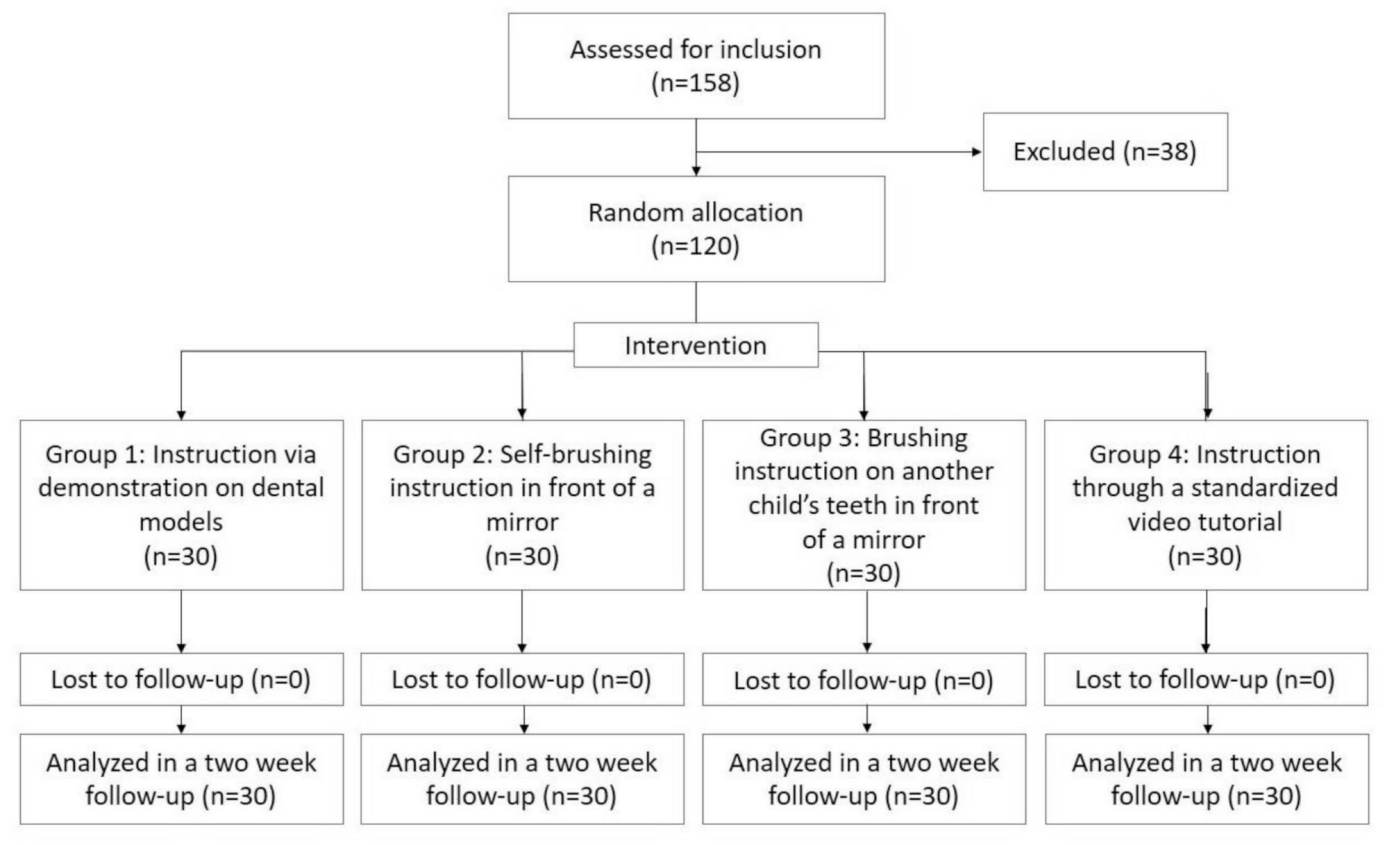
Flow diagram of the participants. From a total of 158 pediatric patients, 120 were selected based on the inclusion criteria and then randomly divided into 4 groups (30 each). Two weeks later, follow-up results were compared in all 4 groups

**Fig. 2:**
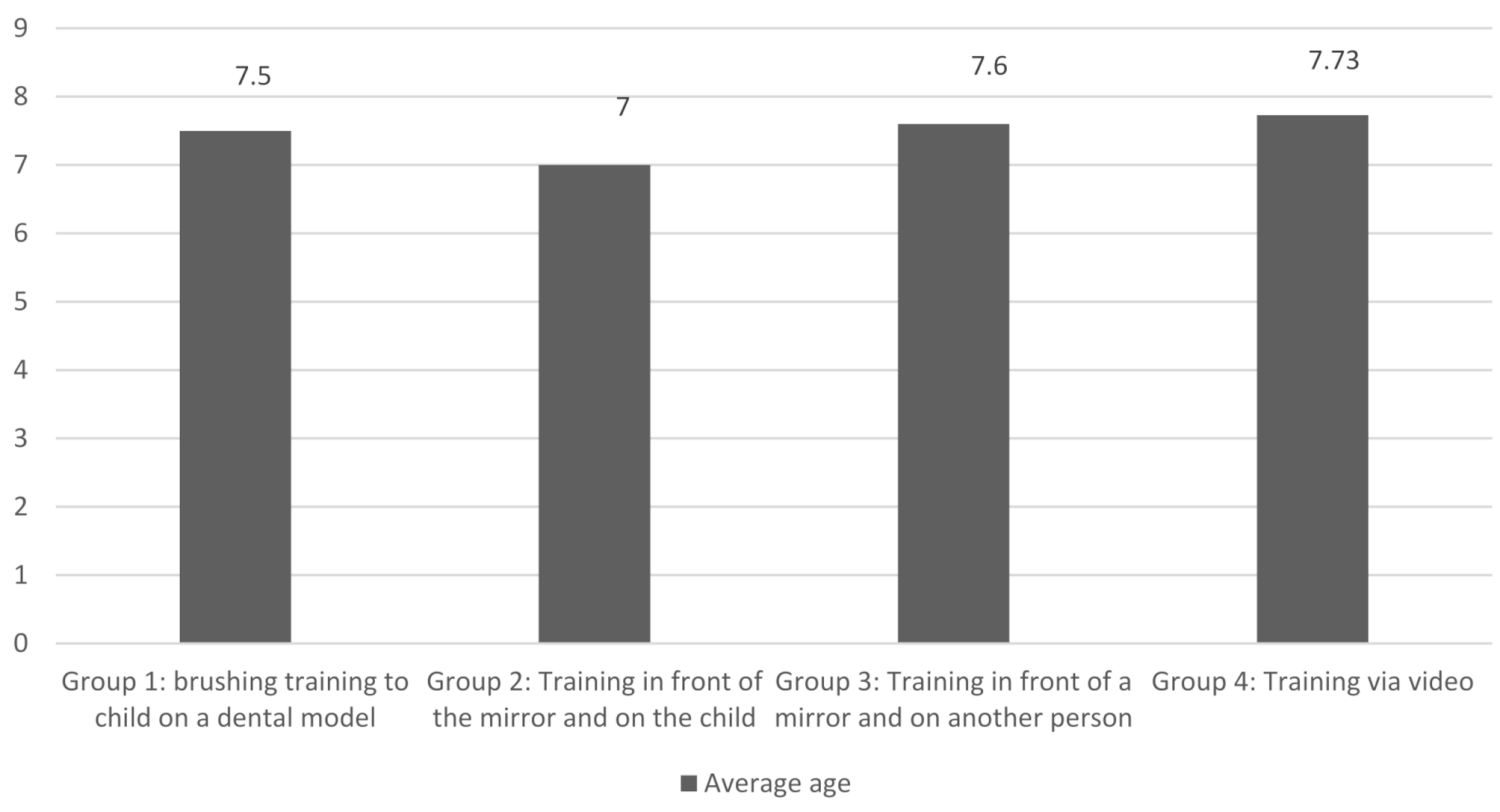
Average age of children referred to the pediatric department of Alborz Dental School in different training groups

The number of 158 children were examined to achieve 120 study participants. No patients have been dropped through follow up (Fig. 1). This study involved four groups of 30 children each (15 boys and 15 girls, aged 6–8 years) attending the pediatric department of the Dental School at Alborz University. There was no significant difference in age among the groups (*P* = 0.63) (Fig. 2).

Table 1 presents the mean O’Leary index values before training, after training, and at the two-week follow-up for each group. The highest mean index before training was observed in Group 1 (74.90 ± 8.12), while the lowest was in Group 2 (70.63 ± 8.90). Differences in the mean O’Leary index before training across the four groups were not statistically significant (*P* = 0.179). However, significant reductions in the O’Leary index were observed across all groups when comparing pre-training values to post-training values and follow-up values (*P* < 0.001).

**Table 1:** The comparison of O’Leary index means before and after training and in the follow-up session by gender in children referred to the pediatric department of Alborz Dental School

		Group 1: dental model		Group 2: participant, mirror		Group3:another person, a mirror		Group 4: educational video	
		mean ± standard deviation	*P*-value*	mean ± standard deviation	*P*-value*	mean ± standard deviation	*P*-value*	mean ± standard deviation	*P*-value*
Before training	girl	72.13 ± 7.88	0.61	72.80 ± 10.49	0.19	75.00 ± 9.35	0.67	77.40 ± 8.60	0.11
	boy	77.67 ± 7.62		68.47 ± 6.62		73.47 ± 10.01		72.53 ± 7.63	
After training	girl	39.87 ± 9.13	0.64	40.13 ± 11.74	0.64	41.20 ± 11.30	0.57	38.67 ± 10.05	0.74
	boy	42 ± 14.96		37.93 ± 13.93		38.67 ± 12.91		39.87 ± 9.78	
Follow-up	girl	37.93 ± 11.75	0.87	42.93 ± 12.50	0.13	39.27 ± 11.00	0.42	40.73 ± 9.65	0.13
	boy	37.13 ± 15.28		36.20 ± 11.23		36.00 ± 10.88		35.00 ± 10.56	

*Based on Independent Samples Test analysis; Level Of significance: 0.05 (*p* ≤ 0.05)

**Table 2:** The comparison of O’Leary index means before and after training and in the follow-up session in children referred to the pediatric department of Alborz Dental School

	Group Educational method	Before training	After training	Follow-up
1	to children on a dental model	74.90 ± 8.12	40.93 ± 12.23	37.53 ± 13.40
2	in front of the mirror and on children	70.63 ± 8.90	39.03 ± 12.70	39.57 ± 12.7
3	in front of the mirror and on another person	74.23 ± 9.55	39.93 ± 11.99	37.63 ± 10.88
4	to children through educational video	70.97 ± 8.36	39.27 ± 9.76	37.87 ± 10.36
	*P*-value*	0.179	0.924	0.898

*Based on ANOVA analysis; Level Of significance: 0.05 (*p* ≤ 0.05)

**Fig. 3:**
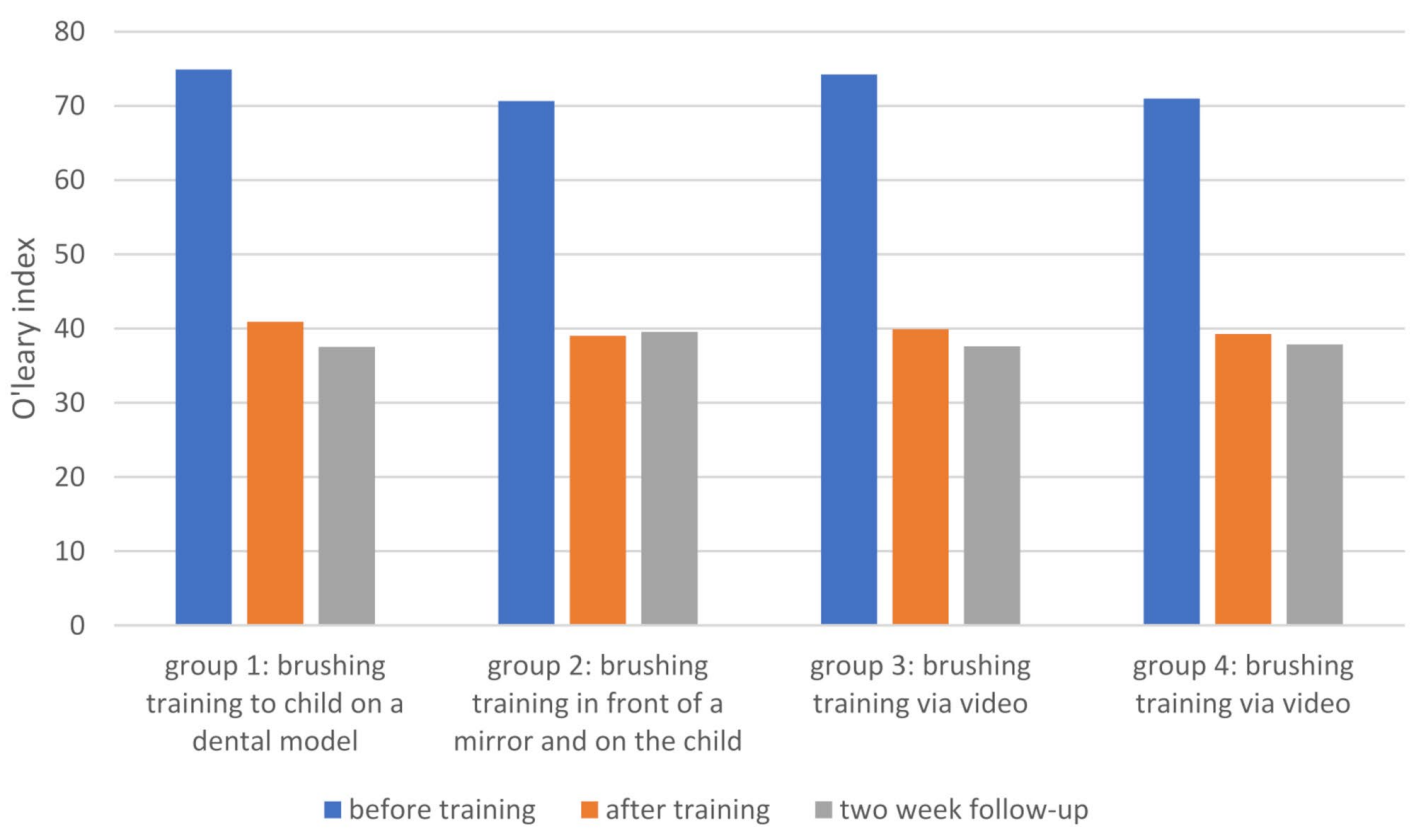
The comparison of O’Leary index means before and after training and in the follow-up session

The greatest reduction in the mean O’Leary index before and after training was observed in Group 1. Despite this, reductions were statistically significant in all groups (*P* < 0.001) (Fig. 3).

Both boys and girls showed decreases in the mean plaque index immediately after training and at the two-week follow-up. The reduction was more pronounced immediately after training, although no significant gender-based differences were observed. Nonetheless, girls demonstrated slightly better results than boys overall (Table 1).

## Discussion

Dental plaque is the primary factor in oral and dental diseases, and regular tooth brushing is the most effective method for its removal [11]. The key outcome of oral hygiene practices is the reduction of plaque, which can be evaluated by measuring residual plaque levels after brushing [26].

A notable strength of this study is its focus on evaluating different educational methods for teaching tooth brushing. While much of the existing research has compared tooth-brushing techniques across various demographics—including age, gender, communities, and geographical conditions—it has often prioritized the influence of toothpaste or toothbrush types on plaque reduction over the learning process itself [27–29]. This study addressed this gap by investigating the most effective instructional methods for children aged 6–8 years.

This specific age group was selected because oral and dental diseases at this stage are largely preventable. Moreover, physical development during this period coincides with the mixed dentition phase, emphasizing the importance of teaching proper oral hygiene techniques. The incomplete mineralization of newly erupted teeth further underscores the need for effective protection and hygiene education [30]. Studies have shown that children of this age often lack foundational knowledge about oral hygiene and appropriate brushing techniques [31]. However, their cognitive abilities and learning capacity make them highly receptive to instruction at this developmental stage [32]. Gauba et al. reported that dental plaque decreases with age as brushing skills improve [33]. By employing a narrow age range in this study, the average age of participants did not significantly influence learning outcomes.

In this study, the O’Leary index was used to evaluate plaque reduction. According to O’Leary et al., this index is a reliable tool for assessing plaque control after oral hygiene education [24]. Previous studies, such as those by Zarabadipor et al., used alternative methods like the Silness and Löe index, which involved grading plaque levels on a scale of 0–3 for each tooth surface and averaging the scores [17]. However, the simplicity and practicality of the O’Leary index make it particularly suitable for pediatric dentistry.

The results of this study demonstrated that tooth-brushing instruction significantly reduced plaque levels in all groups. These findings align with those of Ramezankhani et al., Phuengwongyart et al., Dehdari et al., and Dadipoor et al., who reported similar improvements following educational interventions [20, 21, 24, 25]. Among the four groups, children trained using a dental model showed the greatest reduction in plaque and the most stable results over time.

Srivastava et al. also found that training with dental models yielded the best results when compared to visual media and role modeling in teaching tooth brushing to children [34]. Similarly, Chatterjee et al. reported that dental model instruction resulted in the greatest plaque reduction in adults [35], while Sahaf et al. observed significant improvements in hearing-impaired children who were taught using dental models and guided video training. In their study, the dental model group achieved the highest improvement [36]. However, contrasting findings were reported by Knight et al., who concluded that mirror-based teaching—whether self-directed or using a human model—was superior to methods involving dental casts, dolls, or animal models [37].

Our study also revealed no significant difference between genders. De Farias et al. and Amalia et al., showed better outcomes in girls to their greater attention to training [38, 39]. The reduction in the O’Leary index is also observed in the findings of Buglar et al., Ahn et al., Bhardwaj et al., Chachra et al., Jain et al., Tai et al., and Yi et al. [11, 40–45].

The results of this educational intervention demonstrate its success in improving the oral and dental health of children. Significant reductions in the O’Leary index were observed in all groups after training, corroborating findings from previous studies [20, 22, 24]. The sustained reduction observed during follow-up sessions highlights the importance of teaching effective oral hygiene methods at an early age.

One limitation of this study was the challenge of obtaining parental consent and ensuring participant availability during follow-up sessions. To address this, free dental examinations were offered, and hygiene packages containing toothbrushes and toothpaste were distributed. Future studies should aim to provide long-term evidence of the effectiveness of these instructional methods, as most current research spans less than one year [46].

## Conclusions

The findings of this study demonstrate that educating children on oral and dental hygiene significantly reduces dental plaque and can contribute to long-term improvements in societal oral health. Among the instructional methods evaluated, individual training using a dental model proved to be the most effective and sustainable approach. This method offers great potential for delivering impactful and enduring oral hygiene education, emphasizing its value in preventive dental care programs.

## Electronic supplementary material

Below is the link to the electronic supplementary material.

Supplementary Material 1

Supplementary Material 2

## Data Availability

The datasets used and/or analyzed during the current study are available from the corresponding author upon reasonable request.
