# Supplementary material for: Evaluating the effectiveness of various teaching methods on dental plaque removal in children: a quasi-experimental study
Source: BMC Pediatr. 2025 Feb 12;25:109. doi: 10.1186/s12887-025-05438-6 (PMC11817105; doi:10.1186/s12887-025-05438-6)

| نام و نام خانوادگی:  Name & surname | نام پدر:  Father’s name | تاریخ تولد:   Date of birth |
| --- | --- | --- |
| نام مدرسه :  School name | پایه :  Educational year | کلاس :  class |
| جنسیت:  gender | تحصیلات پدر :  Father’s education | تحصیلات مادر:  Mother’s education |


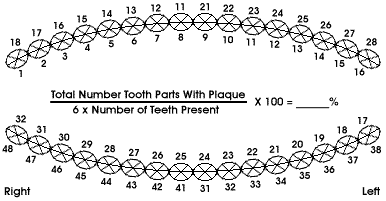

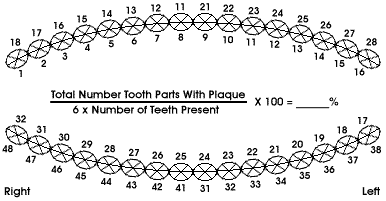

Supplement: Supplementary file 1 — Supplementary Material 1 [file 12887_2025_5438_MOESM1_ESM.docx]
